# Denaturation and solvent effect on the conformation and fibril formation of TGFBIp

**Published:** 2009-12-08

**Authors:** Heather L. Grothe, Morgan R. Little, Angela S. Cho, Andrew J.W. Huang, Ching Yuan

**Affiliations:** 1 Department of Ophthalmology, University of Minnesota, Minneapolis, MN; 2 Department of Ophthalmology and Visual Sciences, Washington University, St. Louis, MO

## Abstract

**Purpose:**

Transforming growth factor beta-induced protein (TGFBIp) aggregates into the phenotypic amyloid fibrils and/or non-amyloid deposits in corneal dystrophies and other disorders. While significant progress has been made in molecular genetics to successfully establish the link between the missense mutations of *TGFBI* and *TGFBIp*-related corneal dystrophies, the underlying mechanism for the abnormal aggregation remains elusive due to the lack of insights into the conformational perturbations induced by mutations. In the present study, we examined the effects of denaturants and a co-solvent on recombinant TGFBIp, with a focus on protein conformational changes and amyloid fibril formation.

**Methods:**

Recombinant TGFBIp was subjected to various spectroscopic studies, such as far-ultraviolet circular dichroism (far-UV CD), intrinsic tryptophan fluorescence and quenching, and 1-anilinonaphthalene-8-sulfonic acid (ANS) fluorescence, under various denaturing conditions (urea and guanidine hydrochloride [GndHCl], acidic pH, and trifluoroethanol [TFE, co-solvent]). A thioflavin T (ThT) fluorescence assay was used to determine the fibril formation of TGFBIp. In addition, a rabbit polyclonal antibody against the oligomer precursors that initiate the formation of amyloid fibrils was also used in dot blot experiments to detect the formation of prefibrillar precursors.

**Results:**

The purified recombinant TGFBIp is in the folded state according to its intrinsic tryptophan fluorescence analyses. A single-step unfolding process was observed in the GndHCl denaturation experiment. Results from far-UV CD, intrinsic tryptophan fluorescence, and ANS fluorescence experiments showed that TFE exerted its solvent effects by initially unfolding and transforming TGFBIp to a β-sheet-enriched conformer at 20%. When increased to 40%, TFE changed TGFBIp into a non-native α-helix conformer. Although GndHCl and TFE led to protein unfolding, enhanced fibril formation could only be observed in the presence of TFE and at acidic pH, according to the ThT fluorescence assays. The paradigmatic protofibrillar TGFBIp oligomers were also detected during the fibril formation by the dot blot experiment.

**Conclusions:**

Our results suggest that protein unfolding may serve as the prerequisite but is not sufficient for the fibrillogenesis. Other factors, such as the solvent used, fragmentation, or pH, may also be crucial for the formation of TGFBIp fibrils.

## Introduction

Pathological protein aggregations, particularly amyloidosis, affect almost all tissues and organs and have been implicated in various human diseases, such as Alzheimer’s disease, Creutzfeldt-Jakob disease, familial amyloidotic polyneuropathy, Parkinson’s disease, and others [1-3]. Corneal amyloidosis disorders, including gelsolin-related lattice corneal dystrophy type II (Finnish-type familial amyloid polyneuropathy type IV), lactoferrin-related familial subepithelial amyloidosis, transforming growth factor beta-induced gene (*TGFBI*)-related corneal dystrophies, and polymorphic amyloid degeneration, are characterized by phenotypic abnormal deposits accompanied by corneal opacities, epithelial abnormalities, pain, and potential loss of vision [4]. In addition to those pathogenic gene mutations that either have systemic effects or specifically affect only corneal tissues, secondary amyloidosis has been reported in various ocular disorders, such as keratitis, chronic post-traumatic inflammation, glaucoma, keratoconus, trichiasis, and even Fuch’s endothelial dystrophies [5-7]. Among these various types of corneal amyloidosis disorders, *TGFBIp*-related corneal dystrophies are perhaps the most well studied.

TGFBIp (the protein product of the *TGFBI* gene, also referred to as keratoepithelin, BIGH3, beta-igh3, βigh3, or RGD-CAP) is a 683-amino acid secretory matrix protein induced by transforming growth factor β-1. It plays essential roles in cell adhesion, differentiation, and tumorigenesis [8-13]. The temporal expression of *TGFBI* during embryogenesis and wound healing suggests its important roles in the development and maintenance of ocular surface integrity [14-16]. Primary sequence analysis reveals that the encoded TGFBIp contains a secretory signal peptide sequence in the NH_2_-terminus, and an arginine-glycine-aspartate (RGD) motif located at the COOH-terminal end, which can mediate cell adhesion and other functions via integrins [17]. In addition, there are four homologous repeats designated as “Fas-like” domains in TGFBIp [18]. The RGD motif in TGFBIp may not be involved in integrin-mediated cell adhesion, but rather in other events, such as apoptosis [19,20]. Mass spectroscopy has indicated that mature TGFBIp from CHO cells does not contain the RGD motif, likely as a consequence of post-translational degradation [21]. Instead, novel motifs (NKDIL and EPDIM) located in the second and fourth Fas-like domains were identified as exerting an integrin-binding capability [10].

The pathogenic roles of mutant TGFBIp in certain types of stromal corneal dystrophies have drawn great attention since TGFBIp’s discovery in 1992 [8]. Genetic analyses [22] have linked several mutations of the *TGFBI* gene to various corneal dystrophies, including Avellino, lattice dystrophies, Reis-Bucklers dystrophies, and granular dystrophies. Currently, more than 30 missense mutations of *TGFBI* have been identified and linked to at least 13 different phenotypes of corneal dystrophies, characterized by the presence of abnormal amyloid fibril and/or non-amyloid deposits in sub-epithelial and stromal layers in the cornea [4,23,24]. Painful corneal erosions often recur, likely due to poor epithelial adhesions. Interestingly, almost all the dystrophy-related mutations are clustered in the first and fourth Fas-like domains (Fas-1 and Fas-4). While the role of genetic predisposition has been well established, it is likely that the genetic mutation is not the only determinant contributing to the amyloid fibril and non-amyloid aggregations in TGFBIp-related corneal dystrophies. The fact that both wild-type and mutant recombinant TGFBIp readily form amyloid fibrils [10] strongly suggests an intrinsic propensity of TGFBIp toward aggregations and a potential pathogenesis mechanism. Previous studies also indicate that TGFBIp contains amyloidogenic motifs that play essential roles in the aggregation mechanism, similar to other disease-related amyloidogenic proteins such as amyloid beta precursor protein or lysozymes. Using synthetic peptide approaches, at least two endogenous amyloidogenic motifs on TGFBIp, spanning from residues 110 to 131 and from 515 to 532, respectively, have been identified [25-27]. However, the role of these amyloidogenic motifs for amyloid fibril formation within the full-length protein context awaits further investigation. In addition, tissue-specific protein or matrix degradation may also play roles in the pathogenesis of the abnormal aggregations of TGFBIp [28].

Protein conformational change and unfolding are proposed to be the prevailing mechanisms for amyloid fibril formation [29]. Studying the molecular properties of disease-related proteins under amyloid-conducive conditions should shed light on the protein aggregation behavior and related pathogenic pathways. Furthermore, studies with recombinant proteins are currently the only avenue for understanding the mechanism of amyloid fibril formation of TGFBIp, since transgenic animal models have failed to manifest corneal dystrophic phenotypes [30]. In this study, we set forth to characterize the conformational properties of TGFBIp with spectroscopic tools, and have investigated the  denaturation profile and the effects of pH and solvent on its conformations and fibril formation.

## Methods

### Materials

All chemical reagents were purchased from Sigma Chemical Co. (St. Louis, MO), if not otherwise specified. Puromycin was purchased from Clontech (Mountain View, CA). Rabbit anti-oligomer polyclonal antibody that recognizes the amino acid sequence-independent oligomeric conformers of various amyloidogenic proteins was purchased from BioSource International (Camarillo, CA). Rabbit anti-TGFBIp polyclonal antibody was custom-made by Bethyl Laboratory (Montgomery, TX). *E. coli*-expressed full-length recombinant TGFBIp was used as the antigen for immunization [31].

### Production of recombinant proteins

The TGFBIp coding sequence from 1 to 641 residues with the RGD motif removed (resembling the matured form of the secreted protein) was PCR-amplified from an I.M.A.G.E. clone (Clone I.D. 2957915; Genbank BE206112), and inserted into pIRES.puro3 for stable expression in mammalian cells (293FT; Invitrogen, Carlsbad, CA). (His)_6_ and Strep II tags were engineered to be expressed right after the encoded signal peptide and in the COOH-terminal end, respectively, to facilitate the subsequent purification. After transfection, cells were maintained in Dulbecco's Modified Eagle Medium plus 10% fetal bovine serum under puromycin selection (3 μg/ml) for two weeks. Individual clones were selected according to their growth rates and TGFBIp expression level (as determined by dot blot experiments). The TGFBIp-expressing clones were further expanded in a serum-free medium system (FreeStyle^TM^ 293, Invitrogen) with reduced puromycin (1 μg/ml). Secreted TGFBIp in the conditioned medium was loaded onto a Ni^+^-NTA column for single-step purification. The purities of recombinant WT TGFBIp were confirmed by SDS-PAGE gels and were routinely higher than 95%. Purified TGFBIp were further concentrated using Centriprep YM-30 (Amicon, Bedford, MA) to the intended concentrations determined by the BCA assay (Pierce, Rockford, IL).

### Fluorescence quenching experiments

Fluorescence studies were performed using a FluoroMax-II spectrofluorometer (Jobin Yvon-SPEX, Edison, NJ). Intrinsic tryptophan fluorescence spectra of recombinant TGFBIp (approximately 0.1 mg/ml in 50 mM Tris-HCl, 150 mM NaCl, pH 7.4) were taken in a 1 cm quartz cuvette at 25 ^o^C. Quenching experiments were carried out by adding aliquots of acrylamide or potassium iodide (KI) stock solutions (5 M) sequentially into recombinant TGFBIp samples. The excitation wavelength was 295 nm (tryptophan) and the emission spectra were scanned from 300 to 500 nm. Sodium thiosulfate (0.1 mM) was added to the KI stock solution to prevent the formation of I_3_^-^, which has a yellowish color that interferes with the spectroscopic measurement. The emission intensities at 330 nm in the absence and in the presence of a quencher (F_o_ and F_OBS_) were used for the Stern-Volmer analyses [32,33]. The data were further corrected for the dilution and the inner filtering effect accordingly [34,35]:

$${\text{F}}_{\text{CORR }}={\text{ F}}_{\text{OBS}}*\text{D}*\text{1}0\text{exp}\left[\left({\text{A}}_{\text{ex}}+{\text{A}}_{\text{em}}\right)/\text{2}\right]$$,

where F_CORR_ and F_OBS_ are the corrected and observed fluorescence, respectively, and D is the dilution factor due to the volume increase from the added quencher; A_ex_ and A_em_ are the absorption of the quencher at the excitation and emission wavelengths, respectively. As the plot of F_0_/F versus acrylamide appeared to be linear (see Results), the two tryptophan residues (TGFBIp contains two tryptophan residues, W68 and W148) likely have heterogeneous fluorescence behaviors, but only differ by a factor of less than 2 within the experimental error [32]. Therefore, the effective quenching constant, K_SV_(eff), was determined *by plotting (F_o_/F-1)/[Q] versus [Q] ([Q], concentration of the quencher) and extrapolating to [Q]=0***,** where the initial slope can be obtained for the value of K_SV_(eff).

### Fluorescence spectroscopy

For the denaturation experiments, the emission maximum (“E_max_”) and intensity-averaged emission maximum (“IAEM,” [λ]=Σ (λ_i_*I_i_) / Σ λ_i_, where I_i_ is the fluorescence intensity at the individual wavelength λ_I_) of tryptophan residues were used as indicators for the unfolding/denaturation of TGFBIp in the presence of *guanidine hydrochloride (GndHCl)* or urea [36]. For Thioflavin T (ThT) assays, 100 μl samples were mixed with 700 μl of 25 μM ThT in 50 mM glycine-NaOH, pH 8.5, and then immediately measured for their ThT fluorescence spectra (excited at 450 nm and scanned from 460 nm to 600 nm). For the pH-dependent amyloid fibril formation, recombinant TGFBIp were mixed with the buffering solution containing 150 mM NaCl and 50 mM of sodium citrate, MES, MOPS, Tris, CHES, and CAPS [27], with pH ranging from 2 to 13. The formed fibrils were further spun down by a TL-100 ultracentrifuge (Beckman Coulter, Fullerton, CA) at 38,500× g (25,000 rpm), washed once in 50 mM Tris-HCl, 150 mM NaCl, pH 7.4 solution and then resuspended in ThT solution for the ThT fluorescence assays to reduce the interference by pH. The fluorescence of 1 anilino-naphthalene-8-sulfonic acid (ANS) was used as a spectroscopic tool to investigate the hydrophobicity of recombinant TGFBIp in various conditions. Samples in 50 μM ANS, 50 mM Tris-HCl, pH 7.4 were measured using an excitation wavelength of 350 nm and scanned from 400 to 600 nm.

### Circular dichroism spectroscopy

The far-UV circular dichroism (CD) spectra were measured by a Jasco J-710 spectropolarimeter (Japan Spectroscopic Co., Tokyo, Japan). Each spectrum was obtained from the average of ten scans with blank-subtraction, smoothed by a binomial smoothing routine, and plotted using Kaleidagraph software (Synergy Software, Reading, PA).

### Electron microscopy

Samples of TGFBIp were incubated at 37 ^o^C in 50 mM Tris-HCl, 150 mM NaCl, pH 7.4 for up to one week. Five microliters of sample were applied onto a carbon-coated formvar copper grid and stained with 2% phosphotungstic acid at pH 7.4 at room temperature for 2 min. The fibrils were examined with a JEOL 1200 transmission electron microscope at the University of Minnesota Characterization facility.

## Results

### Production of recombinant TGFBIp

**Figure 1:**
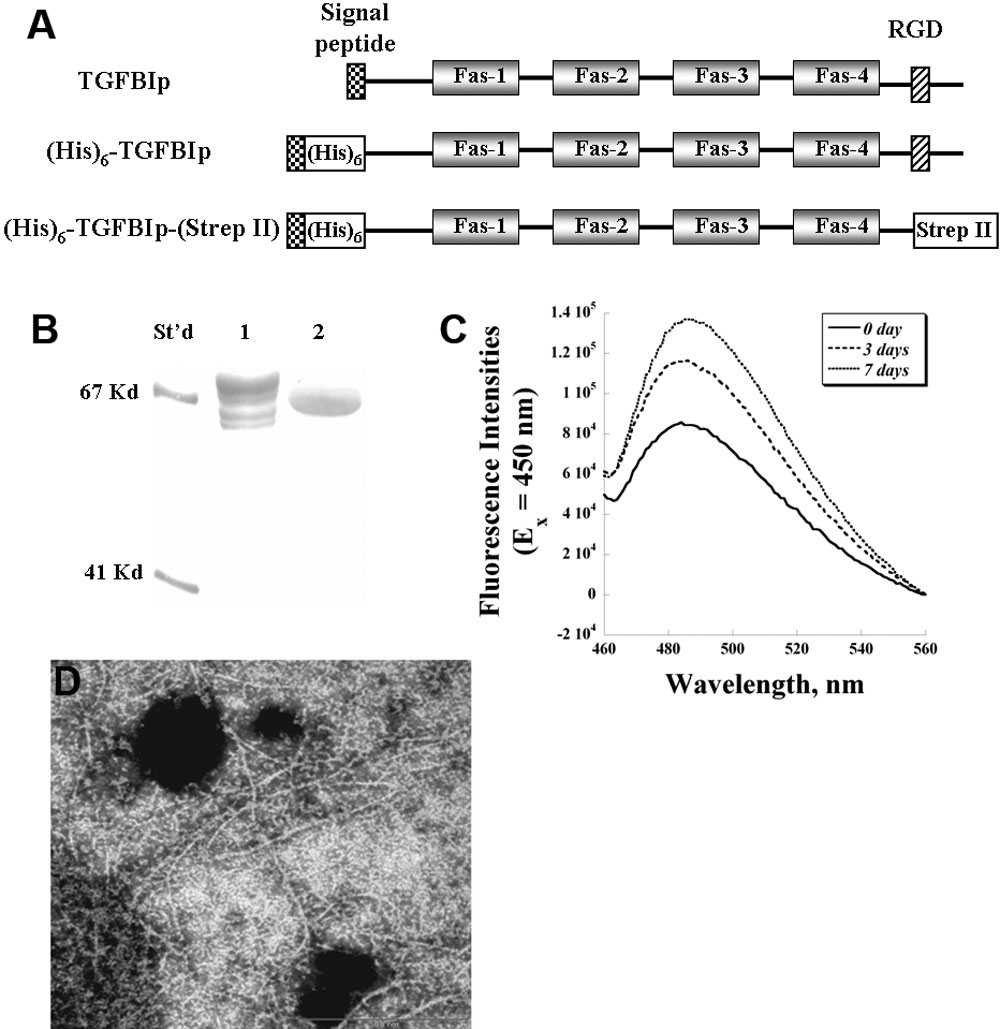
The recombinant transforming growth factor beta-induced protein (TGFBIp) was successfully expressed using mammalian cells. **A**: The *TGFBI* cDNA with various motifs were used for the expression in mammalian cells. "(His)6": polyhistidine tag; "Strep II": strep II tag; "Fas": Fasciclin-like domain; "RGD": arginine-glycine-aspartate motif. **B**: Purified recombinant TGFBIp were analyzed on a 12% SDS-PAGE gel. “St’d”: molecular markers; lane 1: single-tagged (His)_6_-TGFBIp; lane 2: double-tagged (His)_6_-TGFBIp-(Strep II) protein. The amyloid fibril formation of recombinant TGFBIp was obtained after incubation in 1× Tris-buffered saline at 37 °C for one week, as demonstrated by **C**: thioflavin (ThT) fluorescence spectroscopy and by **D**: electron microscopy (75,000× magnification).

In order to obtain highly pure, homogeneous TGFBIp for biochemical and biophysical studies, we have constructed and evaluated various expression vectors (Figure 1A) for recombinant protein production. In contrast to the extensive degradation patterns of recombinant TGFBIp (lane 1, Figure 1B) produced by the single-tagged construct (“[His]_6_-TGFBIp”, Figure 1A), the double-tagged construct with the RGD motif removed (“[His]_6_-TGFBIp-[Strep II]”, Figure 1A) generates a single major protein product (lane 2, Figure 1B). On average, 6–8 mg of purified recombinant TGFBIp was obtained from 1 liter of conditioned medium using a single-step Ni^+^-NTA affinity chromatography, with purities greater than 95%, as demonstrated by SDS-PAGE gel electrophoresis. Previously, WT TGFBIp and peptides have been shown to form fibrils in vitro by various researchers [25-27,37]. Recombinant TGFBIp produced in this study formed fibrils, as demonstrated by the ThT fluorescence assay (Figure 1C) and electron microscopy (Figure 1D) when incubated in the Tris-buffered saline (1× TBS) at 37 ^o^C for one week. Fibrils of 5–10 nm in width and up to several hundred nm in length were observed after extensive incubation at 37 ^o^C (Figure 1D).

### Denaturation profile by urea and GndHCl

**Figure 2:**
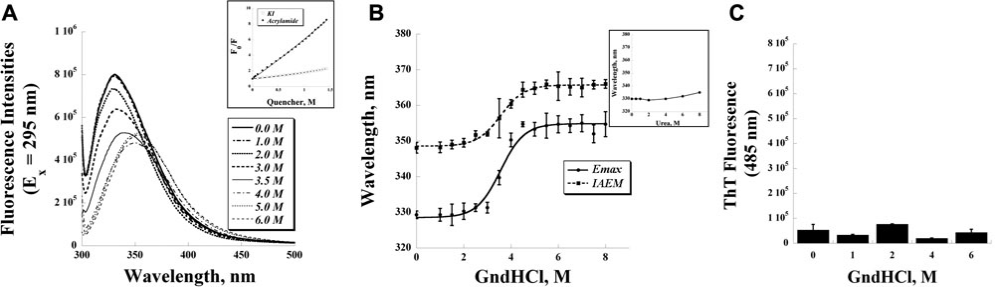
Intrinsic tryptophan fluorescence was used to investigate the quenching and denaturation of  transforming growth factor beta-induced protein (TGFBIp) **A**: Fluorescence traces of TGFBIp (0.1 mg/ml) in guanidine hydrochloride (GndHCl), ranging from 0 to 6 M, were shown. Samples were excited at 295 nm and scanned for the tryptophan emission. Inset: Fluorescence quenching experiments by acrylamide and KI. **B**: The emission maximum (E_max_) and the intensity-averaged emission maximum (IAEM) were obtained by plotting the intrinsic tryptophan fluorescence traces versus GndHCl concentrations. Inset: Denaturation experiments by urea at various concentrations. Plot of the shift of emission maximum versus urea concentration clearly shows that urea fails to unfold TGFBIp, even up to 8 M. **C**: Thioflavin T (ThT) fluorescence  intensity at 485 nm was used to determine the fibril formation of TGFBIp at various GndHCl concentrations (n=3, bar=S.D.).

The intrinsic tryptophan fluorescence of recombinant WT TGFBIp was studied for the denaturation profile using urea and GndHCl. The tryptophan residues display a fluorescence emission peak at 330 nm (E_x_=295 nm) in the native state (Figure 2A, 0.0 M GndHCl), indicating that they are in a relative hydrophobic environment*.* Results from quenching experiments revealed that acrylamide, a non-ionic polar quencher, is able to quench the intrinsic tryptophan fluorescence effectively (Figure 2A, inset). I^-^, on the other hand, failed to produce evident quenching below the concentration of 1 M. Stern-Volmer analyses of the quenching experiments by acrylamide revealed a linear plot, suggesting that the collisional constants of the two tryptophans differ by less than two-fold [32]. The effective collisional quenching constant K_SV_(eff) was determined to be 4.63 M^-1^.

Exposure of tryptophan to the hydrophilic environment during protein unfolding results in a redshift of the emission maximum and a change in the quantum yield. The addition of 1.0 to 6.0 M GndHCl produced a significant redshift of the emission maximum from 330 to 355 nm, along with a significant reduction of fluorescence intensity, as shown in Figure 2A. The concentration of the half-transition by GndHCl denaturation was determined to be approximately 3.5 M (Figure 2B), by both E_max_ and IAEM. The latter index was calculated from the entire spectrum and reflects both the shape and position changes, and is therefore less susceptible to error [36]. The curve-fittings with either indexe also suggested a simple two-state denaturation profile (native and denatured states) by GndHCl. Reduction of the total fluorescence intensity was also observed with increasing GndHCl concentration that approximates a two-state transition (data not shown). Urea at concentrations of up to 8 M, on the other hand, failed to denature recombinant TGFBIp (Figure 2B, inset) and merely produced a 4 nm shift of the emission maximum (from 330 to 334 nm), in contrast to the 25 nm redshift caused by 6 M GndHCl. When WT TGFBIp was incubated with GndHCl at various concentrations that either partially (1 or 2 M) or fully (4 or 6 M) denatured the protein, no fibril formation was observed according to the ThT fluorescence assays (Figure 2C).

### pH effect on fibril formation

**Figure 3:**
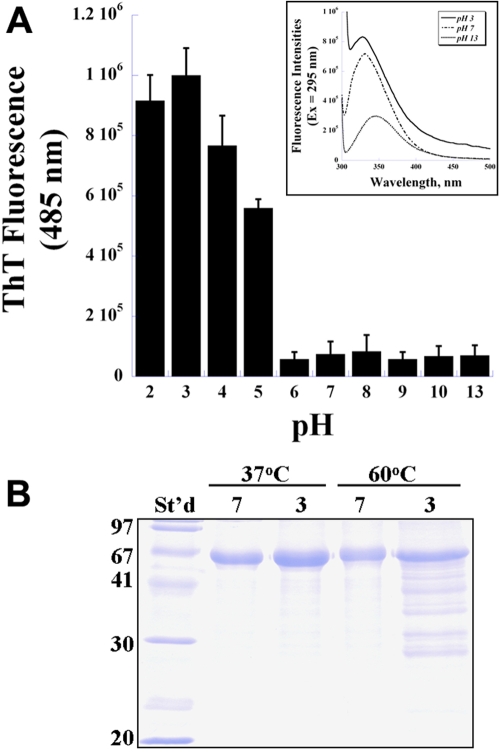
Acidic pH promotes the fibril formation and fragmentation of transforming growth factor beta-induced protein (TGFBIp). **A**: Thioflavin T (ThT) fluorescence intensity at 485 nm was used to determine the fibril formation of TGFBIp at various pH conditions (n=5, bar=S.D.). Inset: the intrinsic tryptophan fluorescence traces of TGFBIp at pH 3, 7, and 13. Inset: Formations of the fibrillar oligomeric precursor of TGFBIp at various TFE concentrations were detected by dot blot experiments. **B**: Chemical fragmentations of TGFBIp under neutral (pH 7) and acidic (pH 3) conditions either at 37 ^o^C or 60 ^o^C, analyzed on a 15% SDS-PAGE gel.

The pH effects on the fibril formation of TGFBIps were investigated by incubating recombinant proteins at various pHs for 24 h at 37 ^o^C. TGFBIps were instantly denatured in alkaline pH (pH 13), as demonstrated by the prominent redshift of the emission maxima of the tryptophan residues (“pH 13”, Figure 3A inset), while aggregations of TGFBIp were promoted in acidic pHs conditions, judging from the elevated light scattering from the baseline (“pH 3” versus “pH 7” in Figure 3A, inset). Significant increases of fibril formations measured by ThT fluorescence assay were noted at conditions below pH 6 (Figure 3A) and reached a plateau at pH 3. On the other hand, the ThT fluorescence intensities remained minimal above pH 6. SDS-PAGE analyses showed that increased fragmentation of TGFBIp occurred at pH 3 after 24 h incubation (37 ^o^C, Figure 3B). Such a degradation in acidic pH was further enhanced by elevating the incubation temperature to 60 ^o^C (Figure 3B).

### Trifluoroethanol promotes the unfolding and fibril formation of recombinant TGFBIp

**Figure 4:**
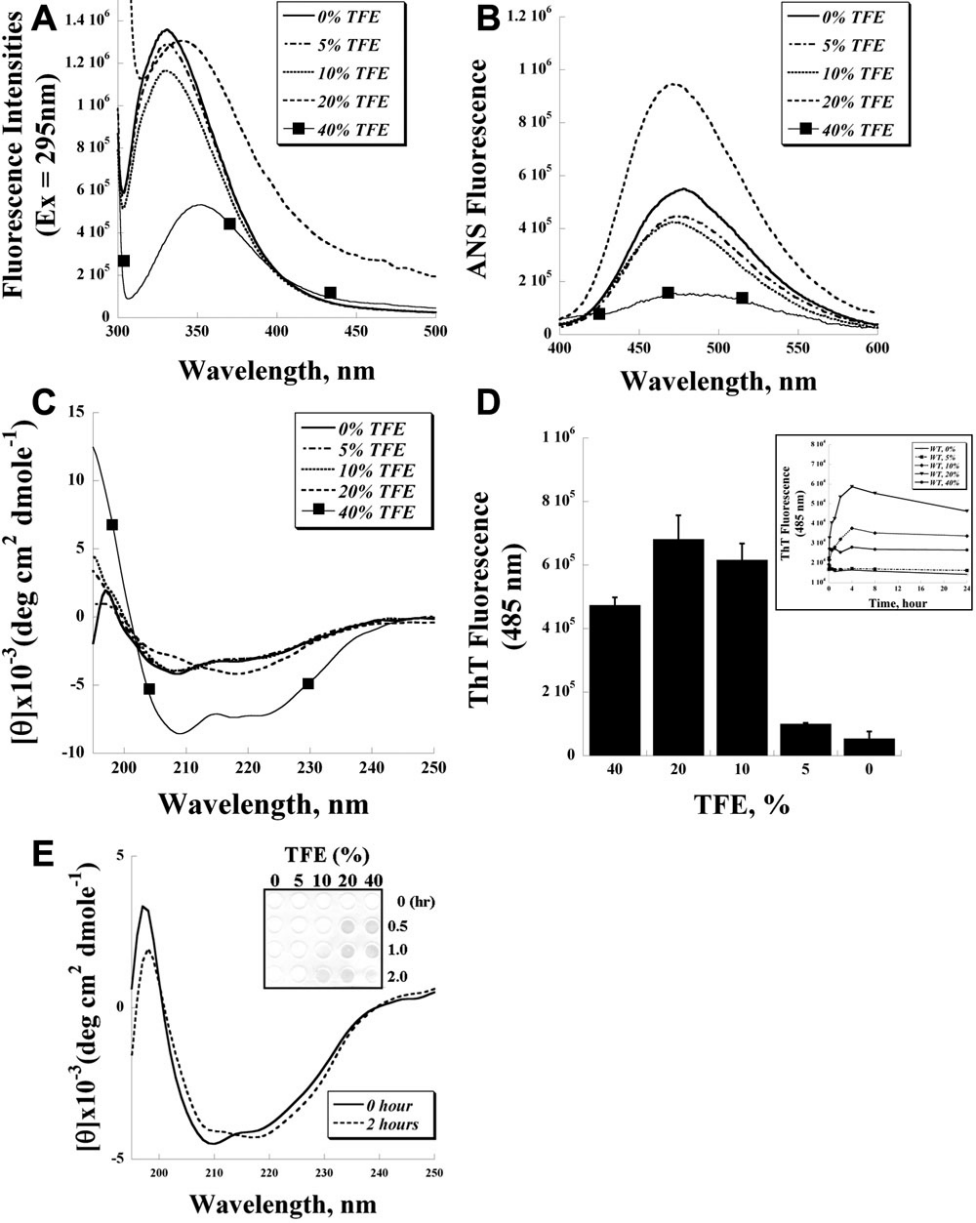
The trifluoroethanol (TFE) effects on the protein conformations of transforming growth factor beta-induced protein (TGFBIp) were studied by **A**: the intrinsic tryptophan fluorescence spectra, B: the 1 anilino-batgakebe-8-sulfonic acid (ANS) fluorescence spectra, and C: the far-ultraviolet circular dichroism (far-UV CD) spectra of recombinant TGFBIp at various TFE concentrations. **D**: Thioflavin T (ThT) fluorescence intensity was used to determine the fibril formation of TGFBIp at various TFE concentrations (n=3, bar=S.D.). Inset: the fibril formation of TGFBIp at various TFE concentrations over a 24 h period. **E**: Far-UV CD spectra were obtained by incubating TGFBIp in 10% TFE for 0 and 2 h. Inset: The dot blot experiment of TGFBIp probed by the anti-oligomer antibody.

As solvation plays essential roles in amyloid fibril formation, we further investigated the solvent effects of trifluoroethanol (TFE) on the conformation and fibril formation of recombinant TGFBIp. The emission maxima of the intrinsic tryptophan fluorescence of TGFBIp were approximately 330 nm in 0–10% TFE (Figure 4A), with the slight reduction of the intensities likely due to the reduced quantum yield in the presence of TFE. At 20% TFE, significant conformational changes of TGFBIp occurred, which is indicated by the shift of the emission maximum toward the red (approximately 348 nm), along with some degree of aggregations reflected by the uprising of the baseline toward the blue (light scattering). At 40% TFE, the tryptophan residues further redshift to approximately 351 nm.

ANS fluoresces to a much higher degree when it binds to exposed hydrophobic patches, and therefore has been used as an indicator for protein unfolding. When ANS was used to probe the unfolding of TGFBIp at various TFE concentrations, minor changes of ANS fluorescence were noted from 0 to 10% TFE (Figure 4B). A prominent ANS increase was noted at 20% TFE, indicating that a conformational change of TGFBIp occurred. At 40% TFE, the ANS fluorescence was significantly reduced.

The far-UV CD spectroscopy (Figure 4C) showed that at sub-denaturation TFE concentrations (0 to 10%), TGFBIp appeared to be in a predominantly α-helix conformation. At 20% TFE, a global conformational change of TGFBIp took place as the α-helix was reduced, with an instant increase of the ellipticity at 218 nm to β-sheet-dominant species. At 40% TFE where the co-solvent forms dynamic clusters, TGFBIp was converted to a non-native α-helix conformer, judging from the prominent 208/222 nm troughs (Figure 4C).

The solvent effects on the fibril formation were studied by incubating TGFBIp with TFE at 37 ^o^C for 24 h and then measured for its ThT fluorescence. The conformation of TGFBIp at 0 or 5% TFE remained unchanged after extensive incubation (48 h, data not shown) and did not produce fibrils in vitro, whereas significant fibril formation was noted in 10 to 20% TFE, with a slight reduction at 40% TFE after 24 h incubation (Figure 4D). Increasing the TFE concentration seemed to affect both the yields and the kinetics of fibril formation (Figure 4D, inset). The fibril formation for TGFBIp at 20% TFE had a fast onset and produced the highest signal at 4 h with a slight decrease subsequently. The 40% TFE produced a quick formation of fibril initially, which was also supported by the results of oligomeric protofibrillar formation (cf., Figure 4E, inset), but the ThT signal remained unchanged subsequently. TGFBIp at 10% TFE showed a slow fibril formation, but reached a level higher than that at 40% TFE.

While 10% TFE did not unfold TGFBIp initially, it eventually induced conformational changes after extended incubation,  as indicated by the CD spec (Figure 4E). Using a polyclonal rabbit antibody that specifically recognizes the universal oligomeric conformers of fibrillar precursors [37], we further demonstrated that TGFBIp formed oligomers in the presence of 10% TFE after 2 h, which could be seen in the dot blot assays used to monitor the oligomeric protofibrillar formation (Figure 4E, inset). On the other hand, no formation of oligomer could be detected at 0 or 5% TFE. Prominent oligomer formations were detected at higher concentrations (20 and 40%) starting 0.5 hour after incubation with TFE. The blots were also probed with the α-TGFBIp antibody to ensure equal loading of proteins in individual wells (data not shown).

## Discussion

Extensive and variable post-translational degradation patterns have been observed for TGFBIp either extracted from tissues, secreted by cultured cells or produced from ectopic mammalian expression systems [19,21,28,38,39], with or without the RGD motif. A comparison study of TGFBIp fragments extracted from normal and diseased corneas (R124C, R124H, and R124L) by 2-D gel electrophoresis further demonstrated distinct degenerative degradations in both the N- and C-termini, suggesting differential proteolysis of mutant proteins [28]. The current construct (“[His]_6_-TGFBIp-[Strep II]”) used in our study has generated a highly purified single TGFBIp protein, compared to the multiple protein products expressed from the (His)_6_-TGFBIp construct or extracted from tissues. The homogeneity and ease of purification of this slightly truncated, matured TGFBIp circumvent the difficulties in obtaining TGFBIp samples with high purity and bioactivity (such as the cell adhesion assay) for biochemical and biophysical experiments. As demonstrated in Figure 1C,D, the purified recombinant TGFBIp can also form amyloid fibrils similar to what other researchers have previously reported [20].

The recombinant TGFBIp displays a two-state denaturation profile by GndHCl, but not by urea. As urea is a nonionic chaotropic agent that denatures proteins by disrupting the hydrophobic interaction, it is likely that additional factors such as electrostatic interaction play significant roles in the folding of TGFBIp. GndHCl, on the other hand, is an ionic chaotropic species that disrupts both hydrophobic and electrostatic interactions, which can fully denature TGFBIp, judging from the emission-maximum shift of the intrinsic fluorescence. TGFBIp contains a cystine-rich domain in the NH_2_-terminus, which potentially can contribute to the folding of TGFBIp via the intramolecular covalent disulfide bond; however, incubating TGFBIp in the presence of urea and reducing agent (dithiothreitol) did not lead to further denaturation (data not shown), which does not support the role of disulfide bond in the folding of TGFBIp. The insights gained from our GndHCl denaturation study will be useful for a future comparison study of dystrophy-causing mutants and WT TGFBIp.

Protein conformational changes and unfolding induced by mutations, oxidation, and other factors have been proposed to be the pathogenesis mechanism for amyloid fibril aggregations [29,40]. One hypothesis for the abnormal aggregation of amyloidogenic proteins is that conformational change and/or protein unfolding exposes the endogenous amyloidogenic regions and facilitates their aggregations into prefibrillar oligomer precursors, fibrils, and eventually amyloid deposits [41]. It has been shown previously that denaturation or partial unfolding by GndHCl can promote amyloid fibril formation in various proteins. For example, GroES (an hsp10 homolog from *E. coli*) readily forms fibrils when partially denatured by GndHCl [42]. However, incubating TGFBIp at partially (1 and 2 M) or fully denaturing (4 M) concentrations did not lead to fibril formation (Figure 2C). Similarly, human and rat hsip10 with sequence homology to GroES failed to form fibrils under GndHCl-perturbed states in a recent study [43]. It is likely that while GndHCl denatures these proteins and exposes their endogenous amyloidogenic regions, it may also disrupt the intermolecular interactions essential for cross-β oligomerization.

Acidic pH conditions promote fibril formation of Aβ peptides, prions, stefin B [44-46], and some non-disease amyloidogenic proteins, as well as TGFBIp (Figure 3A). In addition to the destabilizing effect on protein conformation, low pH conditions can also accelerate the chemical cleavage of peptide bonds of aspartate and glutamate residues [47], which can lead to peptide fragmentation. Such an effect on peptide fragmentation can be further enhanced by high temperatures [48,49]. We observed similar fragmentations and temperature effects with recombinant TGFBIp incubated under low pH conditions (Figure 3B). While extreme pH is rarely encountered under normal physiological conditions (except in the lysozyme), fragmentation of proteins is a key factor in initiating fibril formation in vivo. Peptide fragmentation has been shown to trigger amyloid fibril aggregations of lactoferrin, amyloid-β protein, gelsolin, and other proteins [41,50,51]. Deposits of differentially degraded TGFBIp fragments were noted in the diseased corneas from dystrophic patients, and may likely contribute to the pathogenesis of TGFBIp-related corneal dystrophies [28]. Further study on TGFBIp fragments generated by limited proteolysis or expressed from recombinant sources will help to shed light on the molecular mechanism of TGFBIp amyloidosis.

TFE has been used widely to investigate the solvent effects on amyloidogenic peptides and proteins. With a low dielectric constant, and being mildly more acidic than water, TFE promotes the unfolding and increases the intramolecular hydrogen bonds in proteins [52]. At low concentrations, TFE destabilizes the specific tertiary interactions of native proteins; at higher concentrations, it has been known to stabilize secondary structures (the α-helix) and induce non-native folded states of proteins [53,54]. TFE can also form micelle-like dynamic clusters at higher concentrations (>30%) and modulate the molecular properties of proteins. At the denaturing concentrations, TFE often promotes significant β-sheet formation, and subsequently leads to fibril formation [55]. These effects of TFE on the protein conformation of TGFBIp were also observed. It is clear that TGFBIp formed the non-native α-helical conformers at a high TFE percentage (40%) from CD and intrinsic fluorescence results (Figure 4A,C). At 20% TFE, TGFBIp immediately transformed from an α-helix-enriched native state to a partially denatured state containing predominantly β-sheets (Figure 4C). The transformation not only promoted the aggregating of TGFBIp into the cytotoxic oligomer (a fibrillar precursor, Figure 4E), but also eventually led to prominent fibril formation, according to the ThT fluorescence assays (Figure 4D). Although initially TGFBIp remained in its native state at a sub-denaturation concentration (10%), extended incubation (after 2 h) gradually unfolded TGFBIp and produced oligomers and amyloid fibrils (Figure 4E).

In summary, our study has provided spectroscopic characterizations of recombinant TGFBIp in the presence of denaturants and a co-solvent. Protein unfolding may not be the only determinant for the fibril formation of TGFBIp. Other factors, such as solvents, fragmentation, and pH conditions, also play critical roles. Comparison studies of the molecular properties of WT and dystrophic-related mutant proteins (such as R124C for lattice type I) are currently underway in order to shed light on the pathogenesis mechanism of TGFBIp-related corneal dystrophies.

## References

[1] Glenner GG Amyloid deposits and amyloidosis. The b-fibrilloses (first of two parts). N Engl J Med 1980 302 1283 92 6154243 10.1056/NEJM198006053022305

[2] Kelly JW Alternative conformations of amyloidogenic proteins govern their behavior. Curr Opin Struct Biol 1996 6 11 7 8696966 10.1016/s0959-440x(96)80089-3

[3] Rochet JC Lansbury PT Jr Amyloid fibrillogenesis: themes and variations. Curr Opin Struct Biol 2000 10 60 8 10679462 10.1016/s0959-440x(99)00049-4

[4] Klintworth GK The molecular genetics of the corneal dystrophies, current status. Front Biosci 2003 8 d687 713 12700042 10.2741/1018

[5] Holbach LM, Hinzpeter G, Naumann OH. Kornea und Sklera. In: Naumann GOH, editors. Pathologie des Auges. Berlin, Heidelberg, New York: Springer; 1997. p. 507-692.

[6] Aldave AJ Principe AH Lin DY Yellore VS Small KW Lattice dystrophy-like localized amyloidosis of the cornea secondary to trichiasis. Cornea 2005 24 112 5 15604878 10.1097/01.ico.0000134194.71981.ab

[7] Suesskind D Auw-Haedrich C Schorderet DF Munier FL Loeffler KU Keratoepithelin in secondary corneal amyloidosis. Graefes Arch Clin Exp Ophthalmol 2006 244 725 31 16331487 10.1007/s00417-005-0153-x

[8] Skonier J Neubauer M Madisen L Bennett K Plowman GD Purchio AF cDNA cloning and sequence analysis of beta ig-h3, a novel gene induced in a human adenocarcinoma cell line after treatment with transforming growth factor-beta. DNA Cell Biol 1992 11 511 22 1388724 10.1089/dna.1992.11.511

[9] Hashimoto K Noshiro M Ohno S Kawamoto T Satakeda H Akagawa Y Nakashima K Okimura A Ishida H Okamoto T Pan H Shen M Yan W Kato Y Characterization of a cartilage-derived 66-kDa protein (RGD-CAP/βig-h3) that binds to collagen. Biochim Biophys Acta 1997 1355 303 14 9061001 10.1016/s0167-4889(96)00147-4

[10] Kim JE Kim SJ Lee BH Park RW Kim KS Kim IS Identification of motifs for cell adhesion within the repeated domains of transforming growth factor-β-induced gene, βig-h3. J Biol Chem 2000 275 30907 15 10906123 10.1074/jbc.M002752200

[11] Ahmed AA Mills AD Ibrahim AE Temple J Blenkiron C Vias M Massie CE Iyer NG McGeoch A Crawford R Nicke B Downward J Swanton C Bell SD Earl HM Laskey RA Caldas C Brenton JD The extracellular matrix protein TGFBI induces microtubule stabilization and sensitizes ovarian cancers to paclitaxel. Cancer Cell 2007 12 514 27 18068629 10.1016/j.ccr.2007.11.014 PMC2148463

[12] Ma CRong YRadiloff DRDatto MBCenteno BBao SCheng AWLin FJiang SYeatman TJWang XF Extracellular matrix protein beta ig-h3/TGFBI promotes metastasis of colon cancer by enhancing cell extravasation. Genes Dev 2008 22 308 21 18245446 10.1101/gad.1632008 PMC2216691

[13] Becker J Volland S Noskova I Schramm A Schweigerer LL Wilting J Keratoepithelin reverts the suppression of tissue factor pathway inhibitor 2 by MYCN in human neuroblastoma: a mechanism to inhibit invasion. Int J Oncol 2008 32 235 40 18097563

[14] Rawe IM Zhan Q Burrows R Bunette K Cintron C Beta-ig. Molecular cloning and in situ hybridization in corneal tissues. Invest Ophthalmol Vis Sci 1997 38 893 900 9112985

[15] Takács L Csutak A Balázs E Módis L Jr Berta A Expression of beta ig-h3 is lower than normal in keratoconus corneas but increases with scarring. Cornea 1999 18 599 605 10487436

[16] Schorderet DF Menasche M Morand S Bonnel S Büchillier V Marchant D Auderset K Bonny C Abitbol M Munier FL Genomic characterization and embryonic expression of the mouse Bigh3 (Tgfbi) gene. Biochem Biophys Res Commun 2000 274 267 74 10913330 10.1006/bbrc.2000.3116

[17] Ebel JA, Kuhn K, eds. Molecular Biology Intellingence Unit: Integrin-ligand interaction. Heidelberg, Germany: Springer-Verlag; 1997. p. 1-40.

[18] Kawamoto T Noshiro M Shen M Nakamasu K Hashimoto I Kawashima-Ohya Y Kateo Y Structural and phylogenetic analyses of RGD-CAP/βig-h3, a fasciclin-like adhesion protein expressed in chick chondrocytes. Biochim Biophys Acta 1998 1395 288 92 9512662 10.1016/s0167-4781(97)00172-3

[19] Morand S Buchillier V Maurer F Bonny C Arsenijevic Y Munier FL Schorderet DF Induction of apoptosis in human corneal and HeLa cells by mutated BIGH3. Invest Ophthalmol Vis Sci 2003 44 2973 9 12824240 10.1167/iovs.02-0661

[20] Kim JE Kim SJ Jeong HW Lee BH Choi JY Park RW Park JY Kim IS RGD peptides released from beta ig-h3, a TGF-beta-induced cell-adhesive molecule, mediate apoptosis. Oncogene 2003 22 2045 53 12673209 10.1038/sj.onc.1206269

[21] Skonier J Bennett K Rothwell V Kosowski S Plowman G Wallace P Edelhoff S Disteche C Neubauer M Marquardt H Rodgers J Purchio AF Beta ig-h3: a transforming growth factor-beta-responsive gene encoding a secreted protein that inhibits cell attachment in vitro and suppresses the growth of CHO cells in nude mice. DNA Cell Biol 1994 13 571 84 8024701 10.1089/dna.1994.13.571

[22] Munier FL Korvatska E Djemai A Le Paslier D Zografos L Pescia G Schorderet DF Kerato-epithelin mutations in four 5q31-linked corneal dystrophies. Nat Genet 1997 15 247 51 9054935 10.1038/ng0397-247

[23] Yamamoto S Okada M Tsujikawa M Shimomura Y Nishida K Inoue Y Watanabe H Maeda N Kurahashi H Kinoshita S Nakamura Y Tano Y A kerato-epithelin (betaig-h3) mutation in lattice corneal dystrophy type IIIA. Am J Hum Genet 1998 62 719 22 9497262 10.1086/301765 PMC1376959

[24] Weiss JS Møller HU Lisch W Kinoshita S Aldave AJ Belin MW Kivelä T Busin M Munier FL Seitz B Sutphin J Bredrup C Mannis MJ Rapuano CJ Van Rij G Kim EK Klintworth GK The IC3D classification of the corneal dystrophies. Cornea 2008 Suppl 2 S1 83 19337156 10.1097/ICO.0b013e31817780fb PMC2866169

[25] Schmitt-Bernard CF Chavanieu A Derancour J Arnaud B Demaille JG Calas B Argiles A In vitro creation of amyloid fibrils from native and Arg124cys mutated βigh3110-131 peptides, and its relevance for lattice corneal amyloid dystrophy type I. Biochem Biophys Res Commun 2000 273 649 53 10873659 10.1006/bbrc.2000.2955

[26] Schmitt-Bernard CF Chavanieu A Herrada G Subra G Arnaud B Demaille JG Calas B Argiles A BIGH3 (TGFBI) Arg124 mutations influence the amyloid conversion of related peptides in vitro. Eur J Biochem 2002 269 5149 56 12392546 10.1046/j.1432-1033.2002.03205.x

[27] Yuan C Berscheit HL Huang AJ Identification of an amyloidogenic region on keratoepithelin via synthetic peptides. FEBS Lett 2007 581 241 7 17207483 10.1016/j.febslet.2006.12.019

[28] Korvatska E Henry H Mashima Y Yamada M Bachmann C Munier FL Schorderet DF Amyloid and non-amyloid forms of 5q31-linked corneal dystrophy resulting from kerato-epithelin mutations at Arg-124 are associated with abnormal turnover of the protein. J Biol Chem 2000 275 11465 9 10753964 10.1074/jbc.275.15.11465

[29] Zerovnik E Amyloid-fibril formation. Proposed mechanisms and relevance to conformational disease. Eur J Biochem 2002 269 3362 71 12135474 10.1046/j.1432-1033.2002.03024.x

[30] Kim JE Han MS Bae YC Kim HK Kim TI Kim EK Kim IS Anterior segment dysgenesis after overexpression of transforming growth factor-beta-induced gene, beta igh3, in the mouse eye. Mol Vis 2007 13 1942 52 17982418 PMC2185514

[31] Yuan C Reuters JM Lee L Huang AJW Optimized expression and refolding of human keratoepithelin in BL21(DE3). Protein Expr Purif 2004 35 39 45 15039064 10.1016/j.pep.2003.12.020

[32] Eftink MR Ghiron CA Exposure of tryptophanyl residues in proteins. Quantitative determination by fluorescence quenching studies. Biochemistry 1976 15 672 80 1252418 10.1021/bi00648a035

[33] Eftink MR Ghiron CA Exposure of tryptophanyl residues and protein dynamics. Biochemistry 1977 16 5546 51 921949 10.1021/bi00644a024

[34] Calhoun DB Vanderkooi JM Englander SW Penetration of small molecules into proteins studied by quenching of phosphorescence and fluorescence. Biochemistry 1983 22 1533 9 6342663 10.1021/bi00276a003

[35] Hung HC Chang GG Multiple unfolding intermediates of human placental alkaline phosphatase in equilibrium urea denaturation. Biophys J 2001 81 3456 71 11721007 10.1016/S0006-3495(01)75977-2 PMC1301801

[36] Royer CA Mann CJ Matthews CR Resolution of the fluorescence equilibrium unfolding profile of trp aporepressor using single tryptophan mutants. Protein Sci 1993 2 1844 52 8268795 10.1002/pro.5560021106 PMC2142281

[37] Kayed R Head E Thompson JL McIntire TM Milton SC Cotman CW Glabe CG Common structure of soluble amyloid oligomers implies common mechanism of pathogenesis. Science 2003 300 486 9 12702875 10.1126/science.1079469

[38] Andersen RB Karring H Møller-Pedersen T Valnickova Z Thøgersen IB Hedegaard CJ Kristensen T Klintworth GK Enghild JJ Purification and structural characterization of transforming growth factor beta induced protein (TGFBIp) from porcine and human corneas. Biochemistry 2004 43 16374 84 15610032 10.1021/bi048589s

[39] Kim JE Park RW Choi JY Bae YC Kim KS Joo CK Kim IS Molecular properties of wild-type and mutant betaIG-H3 proteins. Invest Ophthalmol Vis Sci 2002 43 656 61 11867580

[40] Soto C Protein misfolding and disease; protein refolding and therapy. FEBS Lett 2001 498 204 7 11412858 10.1016/s0014-5793(01)02486-3

[41] Dobson CM Protein folding and misfolding. Nature 2003 426 884 90 14685248 10.1038/nature02261

[42] Higurashi T Yagi H Mizobata T Kawata Y Amyloid-like fibril formation of co-chaperonin GroES: nucleation and extension prefer different degrees of molecular compactness. J Mol Biol 2005 351 1057 69 16054644 10.1016/j.jmb.2005.07.006

[43] Yagi H Sato A Yoshida A Hattori Y Hara M Shimamura J Sakane I Hongo K Mizobata T Kawata Y Fibril formation of hsp10 homologue proteins and determination of fibril core regions: differences in fibril core regions dependent on subtle differences in amino acid sequence. J Mol Biol 2008 377 1593 606 18329043 10.1016/j.jmb.2008.02.012

[44] Cardoso I Goldsbury CS Müller SA Olivieri V Wirtz S Damas AM Aebi U Saraiva MJ Transthyretin fibrillogenesis entails the assembly of monomers: a molecular model for in vitro assembled transthyretin amyloid-like fibrils. J Mol Biol 2002 317 683 95 11955017 10.1006/jmbi.2002.5441

[45] Smith DP Jones S Serpell LC Sunde M Radford SE A systematic investigation into the effect of protein destabilisation on beta 2-microglobulin amyloid formation. J Mol Biol 2003 330 943 54 12860118 10.1016/s0022-2836(03)00687-9

[46] Aso Y Shiraki K Takagi M Systematic analysis of aggregates from 38 kinds of non disease-related proteins: identifying the intrinsic propensity of polypeptides to form amyloid fibrils. Biosci Biotechnol Biochem 2007 71 1313 21 17485839 10.1271/bbb.60718

[47] Xie M Schowen RL Secondary structure and protein deamidation. J Pharm Sci 1999 88 8 13 9874696 10.1021/js9802493

[48] Konno T Multistep nucleus formation and a separate subunit contribution of the amyloidgenesis of heat-denatured monellin. Protein Sci 2001 10 2093 101 11567100 10.1110/ps.20201 PMC2374219

[49] Srisailam S Wang HM Kumar TK Rajalingam D Sivaraja V Sheu HS Chang YC Yu C Amyloid-like fibril formation in an all beta-barrel protein involves the formation of partially structured intermediate(s). J Biol Chem 2002 277 19027 36 11877422 10.1074/jbc.M110762200

[50] Sacchettini JC Kelly JW Therapeutic strategies for human amyloid diseases. Nat Rev Drug Discov 2002 1 267 75 12120278 10.1038/nrd769

[51] Nilsson MR Dobson CM In vitro characterization of lactoferrin aggregation and amyloid formation. Biochemistry 2003 42 375 82 12525164 10.1021/bi0204746

[52] Schönbrunner N Wey J Engels J Georg H Kiefhaber T Native-like beta-structure in a trifluoroethanol-induced partially folded state of the all-beta-sheet protein tendamistat. J Mol Biol 1996 260 432 45 8757805 10.1006/jmbi.1996.0412

[53] Mendieta J Folqué H Tauler R Two-phase induction of the nonnative alpha-helical form of beta-lactoglobulin in the presence of trifluoroethanol. Biophys J 1999 76 451 7 9876157 10.1016/S0006-3495(99)77212-7 PMC1302534

[54] Kentsis A Sosnick TR Trifluoroethanol promotes helix formation by destabilizing backbone exposure: desolvation rather than native hydrogen bonding defines the kinetic pathway of dimeric coiled coil folding. Biochemistry 1998 37 14613 22 9772190 10.1021/bi981641y

[55] Yamaguchi K Naiki H Goto Y Mechanism by which the amyloid-like fibrils of a beta 2-microglobulin fragment are induced by fluorine-substituted alcohols. J Mol Biol 2006 363 279 88 16959264 10.1016/j.jmb.2006.08.030

